# Students’ Oral Histories on Family Environment and Alcohol Use: A Qualitative Study

**DOI:** 10.3390/nursrep15110389

**Published:** 2025-11-04

**Authors:** Bruno Pereira da Silva, Gabriel da Silva Brito, Marília Ignácio Espíndola, Clesyane Alves Figueiredo, Cristiano Gil Regis, Maria Giovana Borges Saidel, Débora de Souza Santos, Roberto Ariel Abeldaño Zuñiga, Marluce Mechelli de Siqueira, Manoel Antônio dos Santos, Sandra Cristina Pillon

**Affiliations:** 1 Multidisciplinary Center, Federal University of Acre (UFAC), Cruzeiro do Sul 69890-000, AC, Brazil; cristiano.regis@ufac.br; 2 School of Philosophy, Languages and Literature, and Human Sciences, Federal University of São Paulo (UNIFESP), Guarulhos 02141-000, SP, Brazil; g.brito@unifesp.br; 3 Department of Psychobiology, Federal University of Sao Paulo (UNIFESP), Sao Paulo 04023-062, SP, Brazil; m.espindola@unifesp.br; 4 School of Nursing, State University of Campinas (UNICAMP), Campinas 13000-000, SP, Brazil; c231272@dac.unicamp.br (C.A.F.); mgsaidel@unicamp.br (M.G.B.S.); deborass@unicamp.br (D.d.S.S.); 5 Postgraduate Department, University of Sierra Sur (UNSIS), Miahuatlán de Porfirio Diaz, Oaxaca 70805, Mexico; 6 Helsinki Institute of Urban and Regional Studies, University of Helsinki, 00150 Helsinki, Finland; 7 Centre for Social Data Science, Faculty of Social Sciences, University of Helsinki, 00150 Helsinki, Finland; 8 Department of Nursing, Biomedical Center, Federal University of Espírito Santo (UFES), Vitória 29043-900, ES, Brazil; marluce.siqueira@ufes.br; 9 Faculty of Philosophy, Sciences and Letters of Ribeirão Preto, University of Sao Paulo (USP), Ribeirão Preto 14040-900, SP, Brazil; masantos@ffclrp.usp.br; 10 Department of Psychiatric Nursing and Human Sciences, Ribeirão Preto School of Nursing, University of São Paulo (USP), Ribeirão Preto 14040-092, SP, Brazil; pillon@eerp.usp.br

**Keywords:** alcohol drinking in college, family, family relations, nursing students, nursing

## Abstract

**Objectives**: To investigate family-related factors influencing alcohol use from the perspective of nursing students. **Methods**: A qualitative approach grounded in the Oral History method was employed. Data were collected through interviews with nursing students from a public higher education institution located in the Amazon. Thematic analysis was conducted, supported by a theoretical framework relevant to alcohol consumption. **Results**: Four thematic categories emerged: (1) family inhibition toward alcohol use, (2) implicit prohibition of alcohol within the household, (3) financial dependence on family, and (4) responsibilities associated with adulthood. **Conclusions**: The study highlights the protective role of family structure in shaping young adults’ attitudes toward alcohol. These findings can inform university-level interventions, including: Awareness and education campaigns, Prevention programs, Psychological and psychiatric support services, and Partnerships with local communities.

## 1. Introduction

In recent years, there has been growing research interest in alcohol consumption and its implications among college students [1]. This demographic group, comprising a significant proportion of young adults, has adopted intensive and harmful drinking practices. The binge drinking pattern—characterized by consuming five or more drinks on a single occasion—has been associated with high rates of health problems and frequently appears among causes of mortality in this population [2,3]. According to World Health Organization (WHO) data, approximately 320,000 deaths (representing 10% of total alcohol-related mortality) have been attributed to alcohol-related harm, particularly among youth and adolescents [4]. Alcohol’s effects significantly interfere with students’ academic performance and promote antisocial behaviors, compounding existing health consequences [5].

Beyond these figures, alcohol consumption among university students has been recognized as a global public health challenge. According to the WHO, in 2019, alcohol use accounted for 2.6 million deaths worldwide, including 1.6 million from noncommunicable diseases, 700,000 from injuries, and 300,000 from communicable diseases [6]. International studies show that more than 25% of young adults engage in heavy episodic drinking, with Europe and the Americas reporting the highest prevalence rates [6]. In Sub-Saharan Africa, research among health sciences students in South Africa revealed high lifetime alcohol use (79.1%), past 12-month use (70.2%), and past 30-day use (37.1%), highlighting the vulnerability of university populations in this region [7]. In Brazil, national studies consistently show high prevalence of alcohol consumption among university students, reaching 91.1% in some samples, with almost 40% engaging in binge drinking [8]. At the local level, studies with nursing students have found that more than 90% report alcohol use, underscoring the particular susceptibility of this group due to academic stress and professional exposure to human suffering [9].

Excessive alcohol use among college students has become a serious public health issue, carrying significant implications at individual, family, and societal levels. These consequences manifest through substantial social and healthcare costs associated with alarming rates of interpersonal violence, increased homicide rates, risky sexual behavior, inconsistent condom use, rising infectious disease rates, and traffic accidents—all resulting in significant losses of potential years of life [1,10].

College students have received particular attention due to the vulnerability situations that may emerge during this life stage in the university context. This demographic group, undergoing a biopsychosocial transition process, faces substantial lifestyle changes that often lead to increased engagement in social activities [9]. The transition from school to university life is frequently characterized as a period of intense transformation and growing autonomy, marked by the pursuit of independent living and exploration of new experiences and relationships. However, it often correlates with behaviors that may result in problematic alcohol use, with its well-documented detrimental consequences [5].

Furthermore, college students are particularly susceptible to experimenting with—and in some cases developing—risky alcohol consumption patterns during their academic years. A comprehensive study on alcohol, tobacco, and other substance use patterns, conducted with 12,856 university Brazilian students, revealed concerning consumption rates. Notably, 86% of participants reported lifetime alcohol use, with 78.2% exhibiting low-risk consumption, 19.2% moderate consumption, and 2.6% high-risk consumption. Additionally, binge drinking was widespread, affecting 37.7% of students in the past 12 months and 25.3% in the past 30 days [11].

During the university period—a critical stage of human development—young adults pursue a wide range of experiences, some successful and others less so, with the potential to cause emotional distress regardless of academic challenges [9,12]. Within this context, the establishment of new relationships and the assimilation of values distinct from those transmitted by family traditions become particularly noteworthy. Immersion in unfamiliar social environments expands behavioral repertoires, promoting greater social homogeneity and the possibility of adopting new social roles. Simultaneously, emotional and physical distance from one’s family of origin leads to reduced parental oversight. This distancing, in turn, fosters the development of autonomy and enhances decision-making processes, granting young adults greater confidence and flexibility in navigating available choices [12,13,14].

Research evidence indicates that students’ living conditions are intrinsically linked to alcohol consumption. Those experiencing limited parental oversight—living independently, whether alone, with friends, or in student housing, with minimal family obligations—demonstrate higher propensity for frequent and heavy drinking [1]. A comprehensive study of 3706 students across seven UK universities revealed that living with parents serves as a protective factor against excessive alcohol use [15]. This finding underscores the influential role of family dynamics in shaping student drinking behaviors.

Alcohol remains the most widely consumed psychoactive substance among youth, Nursing students are particularly vulnerable due to the unique demands of their training, which exposes them directly to human suffering and vulnerability [16]. Surprisingly, few studies have examined the family’s role as a potential protective or risk context for alcohol use among college students. Active family supervision and involvement in students’ lives emerge as crucial elements in preventing risky behaviors, including alcohol and other substance use [17].

Family influence extends beyond the immediate environment, significantly impacting young adults’ decision-making process. Studies suggest that family functioning may be pivotal in determining adolescent involvement with psychoactive substances [18]. Among life domains, the family stands out as a context intertwining social dynamics and identity formation. Both protective and risky alcohol-related behaviors have been observed within this sphere, functioning as either risk or protective factors for youth [19].

The family environment, while serving as the foundation for physical and emotional health and transmitting essential values, can also foster harmful habits such as alcohol consumption. Familial issues including lack of communication, intergenerational conflicts, and domestic violence constitute risk factors for alcohol and other psychoactive substance use [19].

In Brazil, studies examining the family as a protective factor against alcohol use among nursing students remain scarce. Most existing research on this topic employs quantitative methodologies and has focused broadly on health sciences students [11,16], emphasizing: Prevalence rates of alcohol use [20], Drinking patterns [11], Risk behaviors [17].

These studies have primarily been conducted in the Southeast and South regions [17,21]. However, family-related factors often appear merely as sociodemographic variables rather than being rigorously assessed as risk or protective factors.

Qualitative studies and research on alcohol consumption among college students in the Amazon are similarly exiguous [17,22,23].

Given that college student alcohol use remains a persistent concern for public policy—and considering the family’s role as the primary social group in psychosocial development—further research is critically needed.

To investigate family-related factors influencing alcohol consumption from the perspective of nursing students.

This study is also informed by theoretical perspectives such as Family Systems Theory and Social Learning Theory, which provide a conceptual basis to understand the ambivalent role of family in alcohol use behaviors. From these perspectives, the family can simultaneously act as a protective factor—by transmitting values, norms, and parental monitoring—and as a risk environment, through modeling of harmful behaviors. This theoretical anchoring strengthens the interpretation of oral histories by situating them within broader frameworks of socialization and intergenerational transmission of behaviors.

## 2. Materials and Methods

**Ethical Considerations**

The study was approved by the Human Research Ethics Committee of the Federal University of São Paulo (Approval Number 569.355), in compliance with the ethical principles for human research established by Resolution 466/2012 of the Brazilian National Health Council. Participants provided written informed consent prior to their involvement in the study.

To ensure participant anonymity, all personal names were replaced with names of rivers from the Amazon region where the participants live. The pseudonyms are: Amônia, Azul, Baje, Crôa, Furquilha, Grajaú, Gregório, Liberdade, Natal, Restauração, São Salvador, and Tejo.

**Theoretical-Methodological Framework and Study Design**

This is a descriptive-exploratory study with a qualitative approach, grounded in the Thematic Oral History method [24,25]. This method relies on recovering participants’ memories through storytelling methodology, drawing on past experiences [24,26]. The oral testimonies of nursing students form the core of this study, enabling qualitative interpretations of family environments and alcohol consumption among university students.

This study adhered to the following qualitative research reporting checklists: Consolidated Criteria for Reporting Qualitative Research (COREQ) [27] and Standards for Reporting Qualitative Research (SRQR) [28]. COREQ is a 32-item guideline divided into three domains, widely used in health research. It aims to enhance the rigor and transparency of qualitative reporting, thereby increasing study credibility. SRQR is a 21-item guideline designed to improve qualitative research quality through standardization criteria [27,28].

**Methodological Procedures**

**Study Setting**

The study was conducted at the Campus Floresta of the Federal University of Acre (UFAC), located in Cruzeiro do Sul, Acre, Brazil.

**Participants**

Participants consisted of 12 undergraduate nursing students from the Campus Floresta, identified through a parent study titled: *“The Meanings of Alcohol Use and Non-Use Among Nursing Students at the Campus Floresta of the Federal University of Acre.”*

Inclusion criteria were as follows: Enrollment in the 6th semester of the Nursing undergraduate program; Completion of coursework in Mental Health Nursing and Psychiatric Nursing; Participation in the project *“Common Mental Disorders, Alcohol, and Tobacco Use Among University Students”.* Thus, sampling was intentional and exhaustive (i.e., all eligible participants were included).

**Recruitment**

Recruitment was facilitated by prior authorization from the Nursing program’s coordination and the cooperation of faculty members, who helped disseminate study information during classes. This approval granted access to: Class attendance lists (based on course offerings); Academic records of students meeting the study criteria for potential phone contact.

**Data Collection and Organization**

Semi-structured Interviews were conducted in 2014, to explore the meanings of alcohol use in the university context and the family environment as a risk or protective factor. A sociodemographic questionnaire was also applied.

The guiding questions were developed based on previous literature on alcohol use among university students and grounded in the principles of oral history methodology, aiming to capture subjective meanings.

Procedure: Pre-contact: Students were initially reached by phone; Scheduling: Interviews were scheduled at the participant’s preferred time and location; Execution: Conducted privately by the study’s first author, using digital audio recording (average duration: 32 min).

**Interview questions**

What is the meaning of alcohol use for society? What is the meaning of alcohol use for you personally? In your view, what factors influence nursing students to use or not to use alcohol?

**Data Analysis**

Analysis followed Meihy and Holanda’s Oral History method [24,25], comprising three stages:

1. Transcription: Verbatim conversion of audio to text (Microsoft Word);
2. Textualization: narratives rewritten in first-person, removal of repetitive terms/questions and logical restructuring to identify core themes;
3. Transcreation: with subjective refinement (e.g., clarifying implied words, describing emotional pauses/silences.

Final texts were validated by each student and thematic categorization involved exhaustive reading of interviews, grouping meaning units by content similarity, reclassification of constitutive elements via analogy and analysis led by the study’s first author.

## 3. Results

Among the interviewed students, nine were women and three were men, ranging in age from 19 to 35 years (mean age: 24), two of whom are 35 years old. Five were married, and seven lived with their families of origin, with nuclear family arrangements being the most common. Four live with their father, mother and siblings and three live with their spouse and children. A summary of participant characteristics is presented in Table 1.

Drawing from the narrative framework of nursing students’ oral accounts, the study reveals that family-related aspects serve as protective factors and help deter alcohol use. The interview analysis identified four thematic categories:

- Family experiences that inhibit alcohol consumption;
- Implicit prohibition of alcohol consumption at home;
- Living with parents and alcohol consumption;
- Being a mother and a nursing student.

The following sections present each category in detail, with illustrative excerpts from participants.

**Family Experiences that Inhibit Alcohol Consumption**

In this thematic category, the family was identified as a protective barrier that discourages children’s proximity to alcoholic beverages. Parental presence was commonly reported as a protective factor against alcohol use, as it distances their children from consumption due to a negative intergenerational history with drinking.

It was also observed that habitual alcohol use by a family member causes distress for both the student and other relatives.

[…] There are some negative role models in my family. Two uncles, who use to live with my grandmother, constantly cause damage to the house when they were inebriated. My father, who I never met, died of alcoholic cirrhosis. Thus, I think drinking is not cool.

(Restauração River)

[…] I’m not saying I’ve never tried, so it’s complicated! I was taught since tender age that alcohol brings no good. Even religion says it. If you drink one dose, you’ll gonna want another one and another, till it turns into dependence. My parents are separated because my father started drinking.

(Tejo River)

**Implicit Prohibition of Alcohol Consumption at Home**

The study also revealed that parents often do not tolerate alcohol consumption by their children at home. It is not uncommon to observe ambivalent discourse within the same family, reflecting broader social contradictions that permeate the familial microcosm. For instance, a father who prohibits his children from drinking at home may habitually consume alcohol himself—even within the household—without consciously recognizing this contradiction.

Although students witness their parents’ alcohol use, they do not feel comfortable drinking at home due to social conventions within parent–child relationships.

[…] My father is the example of someone who he keeps his bottles of alcoholic beverages out of sight.

(Gregório River)

[…] It is controversial that I don’t feel comfortable to grab a drink at home because of my father and my mother. I think: “You can’t do this at home; I don’t do this at home”. So I maintain distance of alcohol when I am at home.

(Gregório River)

For one participant, the model of moral authority inspired by the family acts as an inhibitor of their child’s drinking behavior.

[…] Almost all my classmates are adolescents. Their families have such a great importance for them.

(São Salvador River)

**Living with Parents and Alcohol Consumption**

Residing with family and being financially dependent on parents strengthens student’s internal control over their behavior regarding alcohol consumption. The predominance of students living in the same city as the university campus—while others reside in neighboring municipalities—allows them to commute daily to the university, returning to the family environment at the end of the day.

[…] Nursing students don’t drink much because of their families. They go to college very young and continue living with parents. Being away from family makes it easier to drink.

(Liberdade River)

[…] We are from here, study here and live with our families. I think it’s a protective factor.

(Natal River)

[…] Can you imagine getting home drunk? Things would catch fire next day. If you are still dependent on your father, will you ask him money to drink?

(Baje River)

**Being a Mother and a Nursing Student**

This category revealed that being committed to adult responsibilities inhibits drinking behavior. Emotional maturity is not a linear acquisition that occurs solely during youth and early adulthood. Some participants in this study were married and had already established their own families. Others were in long-term stable relationships (dating, engaged).

[…] In our classroom, there are more family-oriented people, young mothers and older ones. And nobody has this habit of drinking.

(Grajaú River)

[…] The majority of my classmates are married, have children. Some work and don’t have time [for alcohol consumption].

(Tejo River)

[…] My older classmates have jobs, have families, children to raise. I think it wouldn’t be appropriate [alcohol consumption], it would be a bad example for the kids.

(São Salvador River)

## 4. Discussion

These findings can be better understood through the lens of Family Systems Theory and Social Learning Theory, which emphasize the family as a context where values, behaviors, and coping strategies are transmitted intergenerationally. From this perspective, the family simultaneously serves as a protective factor—by establishing norms and providing parental monitoring—and as a potential risk environment when harmful behaviors such as alcohol misuse are modeled.

This study gives a contribution to the limited body of qualitative research on alcohol use among Brazilian university students by highlighting how family arrangements function as protective or risk factors. Our analysis indicates four family-related mechanisms associated with lower alcohol use among nursing students: (i) negative role modelling arising from alcohol-related family violence, (ii) implicit household rules that discourage drinking, (iii) financial dependence that sustains parental oversight, and (iv) adult role responsibilities (e.g., marriage, childcare) that limit opportunities and motivation to drink. These mechanisms align with evidence on alcohol and family violence [18,29], the protective effects of delayed initiation and parental monitoring [30], and the influence of family and peers on youth substance use [29]. Importantly, these mechanisms may not operate uniformly; in some contexts, family exposure to drinking can normalize rather than deter use—heterogeneity we address below.

Participants often recalled traumatic family experiences, such as violence and illness caused by alcohol, which reinforced abstinence and acted as protective factors. This phenomenon illustrates “negative role modelling,” where observing harmful consequences leads individuals to avoid reproducing those behaviors. However, studies show that similar family contexts can also normalize alcohol use, especially when parents or siblings consume regularly [13]. The divergent outcomes underscore the ambivalent role of families—capable of functioning simultaneously as risk and protective environments, depending on the values and coping strategies transmitted to younger generations. Recognizing this ambivalence is essential for designing prevention strategies that move beyond simplistic notions of the family as either protective or harmful, and instead account for its dual and context-dependent influence. This ambivalence is not unique to the Amazonian context. Qualitative studies from other Latin American and Sub-Saharan African countries have also reported families functioning both as inhibitors and facilitators of alcohol use, depending on cultural norms, parental behaviors, and economic circumstances [31,32]. Such cross-cultural resonance suggests that the dual role of the family is a broader phenomenon, though expressed differently according to local contexts.

The accounts also emphasized implicit prohibitions against drinking at home. Even when parents consumed alcohol themselves, participants described unwritten household rules that restricted their own use. This ambivalence reflects broader social contradictions surrounding alcohol in Brazil, where drinking is culturally accepted yet socially regulated within family life. Literature corroborates that delaying initiation—by setting clear boundaries during adolescence—reduces the likelihood of later problematic use. Nevertheless, inhibition based solely on parental presence may lose force in extrafamilial contexts, especially under peer influence [12,14]. Thus, the protective role of implicit prohibition is contingent and may erode when students live away from their families.

Financial dependence emerged as another protective dimension. Reliance on parents for daily expenses and tuition reinforced parental authority and served as a symbolic control mechanism. This aligns with findings that students living independently, whether in dormitories or shared housing, show higher levels of alcohol consumption [19,33]. By contrast, our Amazonian sample largely resided with their families, suggesting that co-residence and economic ties remain central to understanding protective dynamics in this region. Such patterns differ from those reported in the Southeast and South of Brazil, where more students live apart from their families and display higher consumption rates [5,34]. These dynamics suggest that interventions in Amazonian universities should actively involve families, given their central role in regulating student behaviors

Finally, adult responsibilities, such as marriage, childcare, and employment, limited opportunities for social drinking and encouraged moderation. This echoes literature indicating that the assumption of adult roles fosters more cautious health behaviors [35,36]. Importantly, the presence of older and married students in the sample highlights a distinctive profile of Amazonian nursing students, contrasting with studies in other regions where younger, single, and more autonomous students predominate [35,36]. The interaction between age, marital status, and family structure thus helps explain the lower prevalence of alcohol use described in these narratives.

Taken together, these findings contribute to understanding how family arrangements can operate as protective factors in specific sociocultural contexts. They also highlight the need for university-level interventions that consider both family influences and the vulnerabilities that emerge when students move away from parental oversight. Comparative research between different Brazilian regions would help clarify how protective dynamics vary according to living arrangements, economic dependence, and cultural norms. By foregrounding the Amazonian context, our findings invite a broader rethinking of how regional sociocultural specificities shape health behaviors, underscoring the need for tailored, context-sensitive public health policies.

These results have important implications for public health and higher education policies. In Amazonian universities, where family ties remain strong, prevention and intervention strategies could benefit from actively engaging families alongside institutional initiatives. Programs that combine awareness campaigns, psychological and psychiatric support, and community partnerships may amplify protective dynamics while addressing potential risks.

### Limitations and Future Developments

This study has some limitations. The sample was not representative of the “typical” college student population, since most participants were older, married, and all lived with their families rather than independently or in student housing. In addition, eligibility was restricted to students enrolled in a prior project on common mental disorders, alcohol, and tobacco, which may have introduced selection bias. Another limitation is that interviews were conducted in 2014; although social contexts may have changed since then, the family mechanisms identified—such as co-residence, financial dependence, and adult responsibilities—remain relevant to understanding alcohol use among nursing students. However, the pre-test of the questionnaire used was not carried out. Future research should include more diverse and recent samples to verify the persistence of these patterns across different regions and generations. Despite the 2014 data collection, these mechanisms have been consistently described in more recent studies, which justifies the continued relevance of our findings. Additionally, the study relied on a small, homogeneous sample of 12 nursing students from a single program and prior project, which may limit broader representativeness.

In qualitative inquiry, however, the value of findings does not rest on statistical representativeness but on their potential for empirical and naturalistic generalization [37,38,39]. This form of generalization emphasizes transferability, where readers and practitioners assess the applicability of results to other settings with similar social and cultural characteristics. By illuminating family dynamics in the Amazonian context, the study contributes theoretical and practical insights that may resonate in comparable educational and cultural environments, while acknowledging the contextual specificity of the sample.

## 5. Final Considerations

This study, by exploring the protective factors associated with family dynamics in relation to alcohol consumption among nursing students, reveals a significant perception among participants that the family serves as a crucial protective agent in young people’s development—particularly concerning behaviors related to alcohol use.

According to participant accounts, cohabitation with family emerges as a positive factor, playing a fundamental role in discouraging young people from engaging in problematic drinking patterns. Most students still live with and depend financially on their parents, while the assumption of adult roles and responsibilities appears as a key motivational determinant in avoiding alcohol-related behaviors.

A notable aspect in student testimonies is the normalization of alcohol use within family and social settings, reflecting its everyday trivialization. This habit is naturalized as a commonplace behavior, underscoring the need to consider familial and social environmental influences on perceptions of alcohol consumption. Furthermore, the transition from adolescence to adulthood is highlighted as a life-stage that inherently increases susceptibility to experimental and abusive drinking, emphasizing the importance of preventive interventions during this period.

## Figures and Tables

**Table 1:** Characterization of Participants.

Participants	Age	Gender *	Marital Status	Lives with	Number of Siblings	Number of Children
Amônia	35	M	Married	Spouse and children	0	2
Azul	20	F	Single	Father, mother, and siblings	2	1
Baje	20	F	Single	Father, mother, and siblings	1	0
Crôa	22	F	Single	Father and siblings	3	0
Furquilha	19	M	Single	Parents	0	0
Grajaú	35	F	Married	Spouse and child	0	1
Gregório	20	F	Single	Mother and brother	1	0
Liberdade	21	M	Single	Father, mother, and siblings	3	0
Natal	20	F	Single	Father, mother, and sister	1	0
Restauração	28	F	Married	Spouse, sisters-in-law, and son	0	1
São Salvador	29	F	Married	Spouse and daughter	0	1
Tejo	19	F	Married	Spouse	0	0

* M: Male. F: Female.

## Data Availability

Data is contained within the article.

## Public Involvement Statement

No public involvement in any aspect of this research.

## Guidelines and Standards Statement

This manuscript was drafted against the Consolidated Criteria for Reporting Qualitative Research (COREQ) [27] and Standards for Reporting Qualitative Research (SRQR) [28].

## Abbreviations

The following abbreviations are used in this manuscript:

WHO	World Health Organization
UK	United Kingdom
COREQ	Consolidated Criteria for Reporting Qualitative Research
SRQR	Standards for Reporting Qualitative Research
UFAC	Federal University of Acre
MINTER	interinstitutional master’s program
UNIFESP	Federal University of São Paulo
CAPES	Coordination for the Improvement of Higher Education Personnel
EPE	Paulista School of Nursing
M	Male
F	Female
